# Extended Divergence on a Foliation by Deformed Probability Simplexes

**DOI:** 10.3390/e24121736

**Published:** 2022-11-28

**Authors:** Keiko Uohashi

**Affiliations:** Faculty of Engineering, Tohoku Gakuin University, Tagajo 985-8537, Miyagi, Japan; uohashi@mail.tohoku-gakuin.ac.jp

**Keywords:** divergence, exponential family, escort distribution, relative entropy, nonextensive statistics, information geometry, affine geometry

## Abstract

This study considers a new decomposition of an extended divergence on a foliation by deformed probability simplexes from the information geometry perspective. In particular, we treat the case where each deformed probability simplex corresponds to a set of *q*-escort distributions. For the foliation, different *q*-parameters and the corresponding α-parameters of dualistic structures are defined on each of the various leaves. We propose the divergence decomposition theorem that guides the proximity of *q*-escort distributions with different *q*-parameters and compare the new theorem to the previous theorem of the standard divergence on a Hessian manifold with a fixed α-parameter.

## 1. Introduction

In the field of nonextensive statistics, *q*-normal distributions and the generalization, *q*-exponential families, play an important role [1,2,3]. Since Ohara first pointed out the correspondence between the *q*-parameter of nonextensive statistics and the α-parameter of information geometry [4,5], the information geometric structure of *q*-exponential families has been investigated [6,7,8,9,10,11,12,13,14].

On a set of probability distributions, divergences are usually defined for a fixed α-parameter of the dualistic structure. Using those results, we defined an extended divergence on a foliation by sets of probability distributions, setting different α-parameters on each leaf. In particular, we treated a foliation by deformed probability simplexes [15].

In this paper, we also study deformed probability simplexes corresponding to sets of escort distributions with *q*-parameters, which satisfy q=(1−α)/2 for α-parameters of information geometry. We clarify the relationship among affine spaces, affine immersions and the extended divergence more than in our previous paper. A comparison with the extended divergence and the duo Bregman divergence used in machine learning is also described [16].

First, we explain the dualistic structures, α-divergences, and the Tsallis relative entropy on the probability simplex, using the concept of affine geometry and information geometry. The relationship between an α-parameter and the Tsallis *q*-parameter is stated. Next, we describe the dualistic structures and the divergences generated by affine immersions on the deformed probability simplexes corresponding to sets of escort distributions. It also includes topics about Hessian manifolds and their level surfaces. We then define an extended divergence on a foliation by deformed probability simplexes. Finally, we propose a new decomposition of an extended divergence on the foliation.

## 2. The Tsallis Relative Entropy and the Kullback–Leibler Divergence on the Probability Simplex

In this section, we explain dualistic structures, α-divergences, and the Tsallis relative entropy on the probability simplex [4,5,12].

Let $A^{n+1}$ be an (n+1)-dimensional real affine space and $\left\{x^{1},\cdots ,x^{n+1}\right\}$ be the canonical affine coordinate system on $A^{n+1}$, i.e., $\tilde{D}dx=0$, where $\tilde{D}$ is the canonical flat affine connection on $A^{n+1}$. Let $S^{n}$ be a simplex in $A_{+}^{n+1}$ defined by

(1) $$S^{n}=\{p\;|\;p\in A_{+}^{n+1},\sum_{i=1}^{n+1}x^{i}\left(p\right)=1\}.$$

If $x^{1}\left(p\right),\cdots ,x^{n+1}\left(p\right)$ are regarded as probabilities of n+1 states, $S^{n}$ is called the *n*-dimensional probability simplex. Let $\left\{{\bar{p}}^{1},\cdots ,{\bar{p}}^{n}\right\}$ be an affine coordinate system on $S^{n}$ defined by ${\bar{p}}^{i}\left(p\right)=x^{i}\left(p\right)-x^{n+1}\left(p\right)$ for i=1,⋯,n, and

(2) $$\left\{\partial_{1},\cdots ,\partial_{n}\right\},\;\;\;\;\;\;where\;\;\partial_{i}{|}_{p}=\left(\frac{\partial }{\partial x^{i}}-\frac{\partial }{\partial x^{n+1}}\right){|}_{p}\;,\;\;p\in S^{n},$$

be a frame of a tangent vector field on $S^{n}$.

The Fisher metric $g=(g_{ij})$ on $S^{n}$ is defined by

(3) $$g_{ij}\left(p\right)\equiv g\left(\partial_{i},\partial_{j}\right){|}_{p}=\sum_{k=1}^{n+1}x^{k}\left(p\right)\frac{\partial \log x^{k}}{\partial x^{i}}{|}_{p}\frac{\partial \log x^{k}}{\partial x^{j}}{|}_{p}=\frac{1}{x^{i}\left(p\right)}\delta_{ij}+\frac{1}{x^{n+1}\left(p\right)},$$

$$p\in S^{n},\;\;\;\;i,j=1,\cdots ,n,$$

where $\delta_{ij}$ is the Kronecker’s delta. We define an α-connection $\nabla^{\left(\alpha \right)}$ on $S^{n}$ by

(4) $$\nabla_{\partial_{i}}^{\left(\alpha \right)}\partial_{j}=\sum_{k=1}^{n}\Gamma_{ij}^{(\alpha )k}\partial_{k},$$

(5) $$\Gamma_{ij}^{(\alpha )k}{|}_{p}=\frac{1+\alpha }{2}\left(-\frac{1}{x^{k}\left(p\right)}\delta_{ij}^{k}+x^{k}\left(p\right)g_{ij}\left(p\right)\right),\;\;\;\;i,j,k=1,\cdots ,n,$$

where $\delta_{ij}^{k}=1$ if i=j=k, and $\delta_{ij}^{k}=0$ if others. Then, the Levi–Civita connection *∇* of *g* coincides with $\nabla^{\left(0\right)}$. For α∈R, we have

(6) $$Xg\left(Y,Z\right)=g\left(\nabla_{X}^{\left(\alpha \right)}Y,Z\right)+g\left(Y,\nabla_{X}^{\left(-\alpha \right)}Z\right)\;\;\;\;\;\;\mathrm{for}\;X,Y,Z\in X\left(S^{n}\right),$$

where $X(S^{n})$ is the set of all smooth tangent vector fields on $S^{n}$. Then, $\nabla^{\left(-\alpha \right)}$ is called the dual connection of $\nabla^{\left(\alpha \right)}$. For each α, $\nabla^{\left(\alpha \right)}$ is torsion-free and $\nabla^{\left(\alpha \right)}g$ is symmetric. Therefore, the triple $\left(S^{n},\nabla^{\left(\alpha \right)},g\right)$ is a statistical manifold, and $\left(S^{n},\nabla^{\left(-\alpha \right)},g\right)$ the dual statistical manifold of it.

Note that affine connections $\nabla^{\left(1\right)}$ and $\nabla^{\left(-1\right)}$ in Equations (4)–(6) are the dual connection and the canonical connection, respectively.

It is known that when n≥2, the curvature of the statistical manifold $\left(S^{n},\nabla^{\left(\alpha \right)},g\right)$ is a constant value

$$\kappa =\frac{1+\alpha }{2}\;\frac{1-\alpha }{2}=\frac{1-\alpha^{2}}{4}.$$

Therefore, the curvature of the dual statistical manifold $\left(S^{n},\nabla^{\left(-\alpha \right)},g\right)$ is also $\kappa =(1-\alpha^{2})/4$. Iff α=±1, the curvature of $\left(S^{n},\nabla^{\left(\alpha \right)},g\right)$ is zero, and $\left(\nabla^{\left(\alpha \right)},\nabla^{\left(-\alpha \right)},g\right)$ is called the dually flat structure.

For α≠±1, an α-divergence $D^{\left(\alpha \right)}$ on $A_{+}^{n+1}$ is often defined by

(7) $$D^{\left(\alpha \right)}\left(p,r\right)=\frac{4}{1-\alpha^{2}}\left\{\frac{1-\alpha }{2}\sum_{i=1}^{n+1}x^{i}\left(p\right)+\frac{1+\alpha }{2}\sum_{i=1}^{n+1}x^{i}\left(r\right)-\sum_{i=1}^{n+1}x^{i}{\left(p\right)}^{\frac{1-\alpha }{2}}x^{i}{\left(r\right)}^{\frac{1+\alpha }{2}}\right\},$$

$$p,r\in A_{+}^{n+1}.$$

If q=(1−α)/2, it holds that

(8) $$D^{\left(\alpha \right)}\left(p,r\right)=\frac{1}{q}K_{q}\left(p,r\right),\;\;\;\;p,r\in S^{n},$$

for the Tsallis relative entropy $K_{q}$ on $S^{n}$ defined by

(9) $$K_{q}\left(p,r\right)\equiv -\sum_{i=1}^{n+1}x^{i}\left(p\right)\ln_{q}\frac{x^{i}\left(r\right)}{x^{i}\left(p\right)}=\frac{1}{1-q}\left\{1-\sum_{i=1}^{n+1}x^{i}{\left(p\right)}^{q}x^{i}{\left(r\right)}^{1-q}\right\},\;\;\;\;p,r\in S^{n},$$

where $\ln_{q}$ is the q-logarithmic function defined by

(10) $$\ln_{q}x\equiv \frac{x^{1-q}-1}{1-q},\;\;\;\;q\neq 1,\;\;x>0$$

Refs. [1,2]. The Tsallis relative entropy $K_{q}$ converges to the Kullback–Leibler divergence as q→1, because $\lim_{q\to 1}\ln_{q}x=\log x$. In the information geometric view, the α-divergence $D^{\left(\alpha \right)}$ converges to the Kullback–Leibler divergence as α→−1.

For the Tsallis *q*-parameter, the curvature of the statistical manifold $\left(S^{n},\nabla^{\left(\alpha \right)},g\right)$ is κ=q(1−q).

## 3. Divergences Generated by Affine Immersions as Level Surfaces

In this section, we describe the general theory of affine immersions and divergences related to level surfaces of the Hessian domain.

If the Hessian $\tilde{D}d\varphi =\sum_{i,j}\left(\partial^{2}\varphi \right)/\left(\partial x^{i}\partial x^{j}\right)dx^{i}dx^{j}$ of a function φ on a domain $\Omega \subseteq A^{n+1}$ is non-degenerate, the triple $\left(\Omega ,\tilde{D},\tilde{g}=\tilde{D}d\varphi \right)$ is called a Hessian domain. A statistical manifold is said to be flat if the curvature tensor of its affine connection vanishes. A flat statistical manifold is locally a Hessian domain. Conversely, a Hessian domain is a flat statistical manifold [12,17].

In a previous study, we show the following theorem on the level surfaces of a Hessian function.

**Theorem** **1** ([18])**.** *Let M be a simply connected n-dimensional level surface of φ on an (n+1)-dimensional Hessian domain $\left(\Omega ,\tilde{D},\tilde{g}=\tilde{D}d\varphi \right)$ with a Riemannian metric $\tilde{g}$ and suppose that n≥2. If we consider $\left(\Omega ,\tilde{D},\tilde{g}\right)$ a flat statistical manifold, (M,D,g) is a 1-conformally flat statistical submanifold of $\left(\Omega ,\tilde{D},\tilde{g}\right)$, where D and g denote the connection and the Riemannian metric on M induced by $\tilde{D}$ and $\tilde{g}$, respectively.*

Here, “1-conformally flat” represents the characterization of surfaces projected by a flat statistical manifold along dual coordinates. We continue to explain the terms used in Theorem 1 and the outline of the proof.

For α∈R, statistical manifolds (N,∇,h) and $\left(N,\bar{\nabla },\bar{h}\right)$ are α-conformally equivalent if there exists a function ϕ on *N* such that

$$\begin{array}{ccc} \bar{h}\left(X,Y\right) & = & e^{\phi }h\left(X,Y\right),\\ h({\bar{\nabla }}_{X}Y,Z) & = & h\left(\nabla_{X}Y,Z\right)-\frac{1+\alpha }{2}d\phi \left(Z\right)h\left(X,Y\right)\\ & & +\frac{1-\alpha }{2}\left\{d\phi \left(X\right)h\left(Y,Z\right)+d\phi \left(Y\right)h\left(X,Z\right)\right\},\;\;\;\;X,Y,Z\in X\left(N\right). \end{array}$$

If $\left(N,\bar{\nabla },\bar{h}\right)$ is 1-conformally equivalent to a flat statistical manifold (N,∇,h), $\left(N,\bar{\nabla },\bar{h}\right)$ is called a 1-conformally flat statistical manifold. A statistical manifold (N,∇,h) is 1-conformally flat iff the dual statistical manifold $\left(N,\nabla^{\prime },h\right)$ is (−1)-conformally flat [19].

In terms of affine geometry, $\left(N,\nabla^{\prime },h\right)$ and $\left(N,{\bar{\nabla }}^{\prime },h\right)$ are (−1)-conformally equivalent if and only if $\nabla^{\prime }$ and ${\bar{\nabla }}^{\prime }$ are projectively equivalent [20,21].

For an (n+1)-dimensional Hessian domain $\left(\Omega ,\tilde{D},\tilde{g}=\tilde{D}d\varphi \right)$, an *n*-dimensional level surface of φ has the dualistic structure as the statistical submanifold structure. On the other hand, the level surface also has the structure induced by the affine immersion. It is essential for Theorem 1 that the statistical submanifold structure coincides with the dualistic structure by the affine immersion on a level surface of φ.

For $\left(\Omega ,\tilde{D},\tilde{g}=\tilde{D}d\varphi \right)$, let *x* be the canonical immersion of an *n*-dimensional level surface *M* into Ω. Let *E* be a transversal vector field on *M* defined by

(11) $$E=-d\varphi {\left(\tilde{E}\right)}^{-1}\tilde{E},$$

where $\tilde{E}$ is the gradient vector field of φ on Ω defined by

(12) $$\tilde{g}\left(\tilde{X},\tilde{E}\right)=d\varphi \left(\tilde{X}\right),\;\;\;\;\tilde{X}\in X\left(\Omega \right).$$

For an affine immersion (x,E) and the canonical flat affine connection $\tilde{D}$ on $\Omega \subseteq A^{n+1}$, the induced affine connection $D^{E}$, the affine fundamental form $g^{E}$, the shape operator $S^{E}$ and the transversal connection form $\tau^{E}$ on *M* are defined by

(13) $$\begin{array}{ccc} D_{X}Y & = & D_{X}^{E}Y+g^{E}\left(X,Y\right)E, \end{array}$$

(14) $$\begin{array}{ccc} D_{X}E & = & S^{E}\left(X\right)+\tau^{E}\left(X\right)E,\;\;\;\;X,Y\in X\left(M\right). \end{array}$$

See [21,22]. Then, $D^{E}$ and $g^{E}$ coincide with the restricted affine connection of $\tilde{D}$ and the restricted Riemannian metric of $\tilde{g}$, respectively. For the level surface *M*, the transversal connection form satisfies that $\tau^{E}\equiv 0$. Therefore, (x,E) it is called the equiaffine immersion. It is known that a simply connected statistical manifold can be realized in $A^{n+1}$ by a non-degenerate equiaffine immersion iff it is 1-conformally flat [19]. Thus, Theorem 1 holds.

Next, we introduce a divergence on a Hessian domain, treating it as a flat statistical manifold.

The canonical divergence ρ of a Hessian domain $\left(\Omega ,\tilde{D},\tilde{g}=\tilde{D}d\varphi \right)$ is defined by

(15) $$\rho \left(p,r\right)=\varphi \left(p\right)+\varphi^{*}\left(\tilde{\iota }\left(r\right)\right)+\sum_{i=1}^{n+1}x^{i}\left(p\right)x_{i}^{\prime }\left(r\right)\;\;\;\;for\;\;p,r\in \Omega ,$$

where $\tilde{\iota }$ is the gradient mapping from Ω to the dual affine space $A_{n+1}^{*}$, i.e.,

(16) $$x_{i}^{\prime }=x_{i}^{*}\circ \tilde{\iota }=-\frac{\partial \varphi }{\partial x^{i}}\;,$$

and {$x_{1}^{*},\ldots ,x_{n+1}^{*}$} is the dual affine coordinate system of {$x^{1},\ldots ,x^{n+1}$}. The Legendre transform $\varphi^{*}$ of φ is defined by

(17) $$\varphi^{*}\circ \tilde{\iota }=-\sum_{i=1}^{n+1}x^{i}x_{i}^{\prime }-\varphi .$$

See [12].

Let ι be the conormal immersion for the affine immersion (x,E) defined by Equation (11), 12. By the definition of a conormal immersion, ι satisfies that

$$\langle \iota \left(p\right),Y_{p}\rangle =0,\;\;\;\;Y_{p}\in T_{p}M,\;\;\;\;\langle \iota \left(p\right),E_{p}\rangle =1\;\;\;\;\;\;\mathrm{for}\;\;p\in M,$$

where 〈a,b〉 is the pairing of $a\in A_{n+1}^{*}$ and $b\in A^{n+1}$. It is known that the conormal immersion ι coincides with the restriction of the gradient mapping $\tilde{\iota }$ to the level surface *M*.

The next definition is given in relation to affine immersions and divergences.

**Definition** **1** **([** 19 **]).** *Let (N,∇,h) be a 1-conformally flat statistical manifold realized by a non-degenerate affine immersion (v,ξ) into $A^{n+1}$, and w the conormal immersion for v. Then the divergence $\rho_{conf}$ of (N,∇,h) is defined by*

$$\rho_{conf}\left(p,r\right)=\langle w\left(r\right),v\left(p\right)-v\left(r\right)\rangle \;\;\;for\;\;p,r\in N.$$

*The $\rho_{conf}$ definition is independent of the choice of a realization of (N,∇,h).*

The divergence $\rho_{conf}$ is referred to as Kurose geometric divergence in affine geometry and as Fenchel–Young divergence in the machine learning community [23,24]. Since an *n*-dimensional level surface *M* of $\left(\Omega ,\tilde{D},\tilde{g}=\tilde{D}d\varphi \right)$ is a 1-conformally flat statistical manifold realized by a non-degenerate affine immersion (x,E), $\rho_{conf}$ on *M* is as follows:

(18) $$\rho_{conf}\left(p,r\right)=\langle \iota \left(r\right),x\left(p\right)-x\left(r\right)\rangle \;\;\;\mathrm{for}\;\;p,r\in M.$$

Let $\rho_{sub}$ be the restriction of the canonical divergence ρ to (M,D,g) as a statistical submanifold of $\left(\Omega ,\tilde{D},\tilde{g}\right)$. From Equations (15), (17) and (18), the next theorem holds.

**Theorem** **2** ([20])**.** *For a *1*-conformally flat statistical submanifold (M,D,g) of $\left(\Omega ,\tilde{D},\tilde{g}\right)$, two divergences $\rho_{conf}$ and $\rho_{sub}$ coincide.*

## 4. Deformed Probability Simplexes and Escort Distributions Generated by Affine Immersions

In this section, we explain dualistic structures on deformed probability simplexes, which correspond to sets of escort distributions via affine immersion.

We set $p_{i}=x^{i}\left(p\right)$, i=1,⋯,n+1 for $p\in S^{n}$, where $S^{n}$ and $\left\{x^{1},\cdots ,x^{n+1}\right\}$ be the probability simplex and the canonical affine coordinate system on $A^{n+1}$, respectively. For n+1 states $p_{1},\cdots ,p_{n+1}$ on $S^{n}$ and 0<q<1, if each probability $P(p_{i})$ satisfies

(19) $$P\left(p_{i}\right)=\frac{{\left(p_{i}\right)}^{q}}{\sum_{i=1}^{n+1}{\left(p_{i}\right)}^{q}}\;,\;\;\;\;i=1,\cdots ,n+1,$$

the probability distribution P is called the escort distribution [1,2], where ${\left(p_{i}\right)}^{q}$ is $p_{i}$ powered by *q*.

It realizes the dualistic structure of a set of escort distributions via the affine immersion into $A_{+}^{n+1}$ [4,5]. For 0<q<1, let $f_{q}$ be the affine immersion of $S^{n}$ into $A_{+}^{n+1}$ defined by

(20) $$x^{i}\left(f_{q}\left(p\right)\right)=\frac{1}{q}{\left(x^{i}\left(p\right)\right)}^{q},\;\;\;\;i=1,\cdots ,n+1,\;\;\;\;for\;\;p\in S^{n}.$$

Then, the escort distribution P is also represented as follows:

(21) $$P\left(p_{i}\right)=\frac{\theta^{i}}{\sum_{i=1}^{n+1}\theta^{i}},\;\;\;\;\theta^{i}=\frac{1}{q}{\left(p_{i}\right)}^{q},\;\;\;\;\;\;i=1,\cdots ,n+1.$$

For a function $\psi_{q}$ on $A_{+}^{n+1}$ defined by

(22) $$\psi_{q}=\frac{1}{1-q}\sum_{i=1}^{n+1}{\left(qx^{i}\right)}^{\frac{1}{q}},$$

the image $f_{q}\left(S^{n}\right)$ is a level surface of $\psi_{q}$ satisfying $\psi_{q}=1/\left(1-q\right)$. For 0<q<1, the Hessian matrix of the function $\psi_{q}$ is positive definite on $A_{+}^{n+1}$. Then, $\psi_{q}$ induces the Hessian structure $\left(A_{+}^{n+1},\tilde{D},{\tilde{g}}_{q}\equiv \left(\partial^{2}\psi_{q}/\partial x^{i}\partial x^{j}\right)\right)$. By definition

(23) $${\tilde{\Gamma }}_{ijk}=\sum_{l=1}^{n+1}{\tilde{g}}_{q\;kl}\;{\tilde{\Gamma }}_{ij}^{l}=\frac{\partial^{3}\psi }{\partial x^{i}\partial x^{j}\partial x^{k}},\;\;\;\;i,j,k=1,\cdots ,n,$$

(24) $${\tilde{D}}_{\frac{\partial }{\partial x_{i}}}^{\left(\alpha \right)}\frac{\partial }{\partial x_{j}}=\frac{1-\alpha }{2}\sum_{k=1}^{n+1}{\tilde{\Gamma }}_{ij}^{k}\frac{\partial }{\partial x_{k}},\;\;\;\;\alpha =1-2q,$$

the tetrad $\left(A_{+}^{n+1},\tilde{D},{\tilde{D}}^{\left(-1\right)},{\tilde{g}}_{q}\right)$ is the dually flat structure. The connection ${\tilde{D}}^{\left(0\right)}$ coincides with the Levi–Civita connection of the Riemannian metric ${\tilde{g}}_{q}$.

We denote by *D* and $g_{q}$ the restricted $\tilde{D}$ and $\tilde{g_{q}}$ on $f_{q}\left(S^{n}\right)$, and induce the dualistic structure of $\left(f_{q}\left(S^{n}\right),D,g_{q}\right)$ as the submanifold structure of $\left(A_{+}^{n+1},\tilde{D},{\tilde{g}}_{q}\right)$. From the discussion in Section 3, $\left(f_{q}\left(S^{n}\right),D,g_{q}\right)$ coincides with the dualistic structure induced by the equiaffine immersion $\left(f_{q},E_{q}\right)$, where

(25) $$E_{q}\equiv -d\psi_{q}{\left(\tilde{E_{q}}\right)}^{-1}\tilde{E_{q}}$$

for the gradient vector field $\tilde{E_{q}}$ of $\psi_{q}$ on $A_{+}^{n+1}$ defined by

(26) $${\tilde{g}}_{q}\left(\tilde{X},\tilde{E_{q}}\right)=d\psi_{q}\left(\tilde{X}\right)\;\;\;\;\mathrm{for}\;\tilde{X}\in X\left(A_{+}^{n+1}\right).$$

The pullback of $\left(f_{q}\left(S^{n}\right),D,g_{q}\right)$ to $S^{n}$ is (−1)-conformally equivalent to $\left(S^{n},\nabla^{\left(\alpha \right)},g\right)$ defined by Equations (3)–(5). In addition, $\left(f_{q}\left(S^{n}\right),D,g_{q}\right)$ has a constant curvature $\kappa =q\left(1-q\right)=\left(1-\alpha^{2}\right)/4$ [5].

On $\left(f_{q}\left(S^{n}\right),D,g_{q}\right)$, the restricted divergence $\rho_{q}$ from the canonical divergence of $\left(A_{+}^{n+1},\tilde{D},{\tilde{g}}_{q}\right)$ coincides with the geometric divergence by Equation (18) from the affine immersion $\left(f_{q},E_{q}\right)$. For an affine coordinate system $\left\{x_{1}^{\prime },\cdots ,x_{n+1}^{\prime }\right\}$ on $A^{n+1}$ defined by

(27) $$x_{i}^{\prime }=-\frac{\partial \psi_{q}}{\partial x^{i}}=-\frac{1}{1-q}{\left(qx^{i}\right)}^{\frac{1-q}{q}}\;,$$

the divergence $\rho_{q}$ of $\left(f_{q}\left(S^{n}\right),D,g_{q}\right)$ is described as

(28) $$\rho_{q}\left(a,b\right)=\sum_{i=1}^{n+1}x_{i}^{\prime }\left(b\right)\left(x^{i}\left(a\right)-x^{i}\left(b\right)\right),\;\;\;\;\;\;a,b\in f_{q}\left(S^{n}\right).$$

In addition, the pullback divergence of $\rho_{q}$ to $S^{n}$ coincides with $D^{\left(\alpha \right)}$ and the Tsallis relative entropy $K_{q}$ [4].

At the end of this section, we mention the divergence of $\left(A_{+}^{n+1},\tilde{D},{\tilde{g}}_{q}\right)$. By Equation (17), the Legendre transform $\psi_{q}^{*}$ of $\psi_{q}$ is

(29) $$\psi_{q}^{*}\left(x^{\prime }\left(a\right)\right)=-\psi_{q}\left(a\right)+\sum_{i=1}^{n+1}x^{i}\left(a\right)x_{i}^{\prime }\left(a\right),\;\;\;\;a\in A_{+}^{n+1}.$$

By Equations (15) and (16), the canonical divergence $\rho_{q}$ of $\left(A_{+}^{n+1},\tilde{D},{\tilde{g}}_{q}\right)$ is defined by

(30) $$\rho_{q}\left(a,b\right)=\psi_{q}\left(a\right)-\psi_{q}\left(b\right)+\sum_{i=1}^{n+1}x_{i}^{\prime }\left(b\right)\left(x^{i}\left(a\right)-x^{i}\left(b\right)\right),\;\;\;\;a,b\in A_{+}^{n+1}\;,$$

represented by the same symbol $\rho_{q}$ of $\left(f_{q}\left(S^{n}\right),D,g_{q}\right)$.

## 5. Extended Divergence on a Foliation by Deformed Probability Simplexes

Previous sections described the divergence for each fixed *q* and each fixed α. This section defines an extended divergence on a foliation by deformed probability simplexes $\left(f_{q}\left(S^{n}\right),D,g_{q}\right)$ for all 0<q<1, and shows the divergence decomposition theorem. The contents of our paper [15] are included but are explained in detail by the setting of affine geometry.

To give the proximity of *q*-escort distributions with different *q*-parameters, we define an extended divergence on a foliation by deformed probability simplexes as follows.

**Definition** **2.** *Let $S_{fol}=\cup_{0<q<1}\;f_{q}\left(S^{n}\right)=\{p\;|\;p\in A_{+}^{n+1},\;\sum_{i=1}^{n+1}x^{i}\left(p\right)>1\}$, which corresponds to a foliation $F=\{f_{q}\left(S^{n}\right)|0<q<1\}$. We call a function $\rho_{fol}$ on $S_{fol}\times S_{fol}$ defined by Equation (31) an extended divergence on a foliation by deformed probability simplexes.*

(31) $$\rho_{fol}\left(a,b\right)\equiv \psi_{q(a)}\left(a\right)-\psi_{q(b)}\left(b\right)+\sum_{i=1}^{n+1}x_{i}^{\prime }\left(b\right)\left(x^{i}\left(a\right)-x^{i}\left(b\right)\right)$$

$$for\;\;a\in f_{q(a)}\left(S^{n}\right),\;\;b\in f_{q(b)}\left(S^{n}\right),\;\;\;\;\;\;0<q\left(a\right)<1,\;\;\;0<q\left(b\right)<1.$$

The *i*-th component of the conormal immersion of $\left(f_{q},E_{q}\right)$ is $-\partial \psi_{q}/\partial x^{i}$. By the right-hand side of Equation (27), the dual coordinate of *b*, denoted by $x^{\prime }\left(b\right)$, satisfies that

$$-x^{\prime }\left(b\right)\equiv \left(-x_{1}^{\prime }\left(b\right),\cdots ,-x_{n+1}^{\prime }\left(b\right)\right)\in f_{1-q(b)}\left(S^{n}\right).$$

Therefore, we consider $f_{1-q}\left(S^{n}\right)$ as the dual simplex of $f_{q}\left(S^{n}\right)$ for 0<q<1. As q=1/2, $f_{q}\left(S^{n}\right)$ is self dual [4]. Note that the *i*-th component of the dual coordinate of *b* is denoted by $\eta_{i}\left(b\right)=-x_{i}^{\prime }\left(b\right)=\left(\partial \psi_{q}/\partial x^{i}\right){|}_{b}$ in [15].

On the extended divergence, the next proposition holds.

**Proposition** **1.** *An extended divergence $\rho_{fol}$ on $S_{fol}$ of satisfies that:*

*(i) If $a,b\in f_{q(a)}\left(S^{n}\right)$,*

$$\rho_{fol}\left(a,b\right)=\rho_{q(a)}\left(a,b\right)=D^{\left(\alpha (a)\right)}\left(f_{q(a)}^{-1}\left(a\right),f_{q(a)}^{-1}\left(b\right)\right),$$

*where* $\rho_{q}$ *is the divergence of* $\left(f_{q}\left(S^{n}\right),D,g_{q}\right)$ *by Equation (28),* $D^{\left(\alpha \right)}$ *is an α-divergence defined by Equation (7), and* α(a)=1−2q(a) *.*

*(ii) In the case of q(a)≥q(b),*

$$\rho_{fol}\left(a,b\right)\geq 0\;\;\;\;for\;\;\left(a,b\right)\in S_{fol}\times S_{fol},$$

*and if and only if a=b,*

$$\rho_{fol}\left(a,b\right)=0.$$

**Proof.**  If $a,b\in f_{q(a)}\left(S^{n}\right)$, $\psi_{q(a)}\left(a\right)=\psi_{q(b)}\left(a\right)=\psi_{q(b)}\left(b\right)$. By Equations (28) and (31),

$$\rho_{fol}\left(a,b\right)=\sum_{i=1}^{n+1}x_{i}^{\prime }\left(b\right)\left(x^{i}\left(a\right)-x^{i}\left(b\right)\right)=\rho_{q(a)}\left(a,b\right).$$

Then, (i) holds. If 1>q(a)≥q(b)>0, it holds that $\psi_{q(a)}\left(a\right)\geq \psi_{q(b)}\left(b\right)$ because

(32) $$\psi_{q(a)}\left(a\right)=\frac{1}{1-q(a)},\;\;\;\;\psi_{q(b)}\left(b\right)=\frac{1}{1-q(b)}$$

are induced by the definition of $f_{q}\left(S^{n}\right)$. In addition, $f_{q(a)}\left(S^{n}\right)$ and $f_{q(b)}\left(S^{n}\right)$ are convex surfaces centered on the origini of $A_{+}^{n+1}$, and the surfaces $f_{q(a)}\left(S^{n}\right)$ closer to the origin than $f_{q(b)}\left(S^{n}\right)$. Then, $\sum_{i=1}^{n+1}x_{i}^{\prime }\left(b\right)\left(x^{i}\left(a\right)-x^{i}\left(b\right)\right)\geq 0$. Thus, (ii) holds. □

We define the *extended dual divergence* $\rho_{fol}^{*}$ of $\rho_{fol}$ as follows;

(33) $$\rho_{fol}^{*}\left(a,b\right)\equiv \psi_{q(a)}^{*}\left(x^{\prime }\left(a\right)\right)-\psi_{q(b)}^{*}\left(x^{\prime }\left(b\right)\right)+\sum_{i=1}^{n+1}x^{i}\left(b\right)\left(x_{i}^{\prime }\left(a\right)-x_{i}^{\prime }\left(b\right)\right)\;\;\;\;$$

$$for\;\;a\in f_{q(a)}\left(S^{n}\right),\;\;b\in f_{q(b)}\left(S^{n}\right),\;\;\;\;0<q\left(a\right)<1,\;\;\;0<q\left(b\right)<1,$$

where $\psi_{q}^{*}$ is the Legendre transform of $\psi_{q}$ for 0<q<1. Then, the following holds.

**Proposition** **2.** The functions $\rho_{fol}$ and $\rho_{fol}^{*}$ satisfy that

(34) $$\rho_{fol}^{*}\left(b,a\right)=\rho_{fol}\left(a,b\right)\;\;\;\;\;\;for\;\;a\in f_{q(a)}\left(S^{n}\right),\;\;b\in f_{q(b)}\left(S^{n}\right).$$

**Proof.**  By the definition of the Legendre transform, we have

$$\begin{array}{ccc} \rho_{fol}^{*}\left(b,a\right) & = & \psi_{q(b)}^{*}\left(x^{\prime }\left(b\right)\right)-\psi_{q(a)}^{*}\left(x^{\prime }\left(a\right)\right)+\sum_{i=1}^{n+1}x^{i}\left(a\right)\left(x_{i}^{\prime }\left(b\right)-x_{i}^{\prime }\left(a\right)\right)\\ & = & -\psi_{q(b)}\left(b\right)-\sum_{i=1}^{n+1}x^{i}\left(b\right)x_{i}^{\prime }\left(b\right)\\ & & +\psi_{q(a)}\left(a\right)+\sum_{i=1}^{n+1}x^{i}\left(a\right)x_{i}^{\prime }\left(a\right)+\sum_{i=1}^{n+1}x^{i}\left(a\right)\left(x_{i}^{\prime }\left(b\right)-x_{i}^{\prime }\left(a\right)\right)\\ & = & \psi_{q(a)}\left(a\right)-\psi_{q(b)}\left(b\right)+\sum_{i=1}^{n+1}x_{i}^{\prime }\left(b\right)\left(x^{i}\left(a\right)-x^{i}\left(b\right)\right)\\ & = & \rho_{fol}\left(a,b\right). \end{array}$$

□

The extended divergence is related to the duo Bregman (pseudo-)divergence, where the parameters also define the convex functions [16]. To work with the entire parametrized probability distribution families and to explore the application of divergences, we must investigate their relationship.

## 6. Decomposition of an Extended Divergence

In this section, we explain the orthogonal foliation of F. Next, we give a decomposition of an extended divergence along the orthogonal leaf and the original leaf.

For the foliation $F=\{f_{q}\left(S^{n}\right)|0<q<1\}$, we consider the flow on $S_{fol}$ defined using the following equation.

(35) $$\frac{dx_{i}^{\prime }}{dt}=x_{i}^{\prime }\;,\;\;\;\;i=1,\cdots ,n+1,$$

where a function $x_{i}^{\prime }$ on $S_{fol}$ takes the *i*-th component of the dual coordinate on $f_{q}\left(S^{n}\right)$ as Equation (27) for each 0<q<1. An integral curve of Equation (35) is orthogonal to $f_{q}\left(S^{n}\right)$ for each *q* with respect to the pairing 〈,〉. The set of integral curves becomes the orthogonal foliation of F. We denote it by $F^{⊥}$.

Translating into the primal coordinate system, we have the next equation.

(36) $$\frac{dx^{i}}{dt}={\tilde{E}}^{i},\;\;\;\;\;\;i=1,\cdots ,n+1,\;\;\;\;\mathrm{on}\;\;S_{fol},$$

(37) $$\tilde{E}={\tilde{E}}_{q}^{i}=\sum_{j=1}^{n+1}g_{q}^{ij}\frac{\partial \psi_{q}}{\partial x^{j}}\;\;\;\;\mathrm{on}\;\;f_{q}\left(S^{n}\right),$$

where $\left(g_{q}^{ij}\right)$ is the inverse matrix of $\left(g_{q\;ij}\right)$. The right-hand side of Equation (37) is calculated using Equations (11) and (12) for $\psi_{q}$. A leaf of $F^{⊥}$ is an integral curve of the vector field $\tilde{E}$ that takes the value ${\tilde{E}}_{q}$ on $f_{q}\left(S^{n}\right)$ for each *q*.

The following theorem is on the decomposition of the extended divergence.

**Theorem** **3.** *Let $S^{n}$ be the probability simplex, and $\left(f_{q}\left(S^{n}\right),D,g_{q}\right)$ the 1-conformally flat statistical manifold generated by the affine immersion $\left(f_{q},E_{q}\right)$, where $f_{q}$ is defined as*

(38) $$x^{i}\left(f_{q}\left(p\right)\right)=\frac{1}{q}{\left(x^{i}\left(p\right)\right)}^{q},\;\;\;\;i=1,\cdots ,n+1,\;\;\;\;for\;\;p\in S^{n},$$

*$\psi_{q}\equiv 1/\left(1-q\right)\sum_{i=1}^{n+1}{\left(qx^{i}\right)}^{1/q}$, $E_{q}\equiv -d\psi {\left({\tilde{E}}_{q}\right)}^{-1}{\tilde{E}}_{q}$, ${\tilde{E}}_{q}^{i}\equiv \sum_{j=1}^{n+1}g_{q}^{ij}\;\partial \psi_{q}/\partial x^{j}$, and $g_{q}$ is the restriction of $\left(g_{q\;ij}\right)=Dd\psi_{q}$ to $f_{q}\left(S^{n}\right)$. Let $a,b\in f_{q(a)}\left(S^{n}\right)$, 0<q(a)<1, and $c\in S_{fol}\equiv \cup_{0<q<1}\;f_{q}\left(S^{n}\right)$. If there exists an orthogonal leaf $L^{⊥}\in F^{⊥}$ that includes b and c, we have*

(39) $$\rho_{fol}\left(a,c\right)=\mu \;\rho_{fol}\left(a,b\right)+\rho_{fol}\left(b,c\right),\;\;\;\;x^{\prime }\left(c\right)=\mu \;x^{\prime }\left(b\right),\;\;\mu >0,$$

*where $x^{\prime }\left(\cdot \right)$ is the dual coordinate of $f_{q}\left(S^{n}\right)$ for each q.*

**Proof.**  From $a,b\in f_{q(a)}\left(S^{n}\right)$, it holds that $\psi_{q(a)}\left(a\right)=\psi_{q(b)}\left(b\right)$, where q(b)=q(a). By the definition in Equations (22) and (23), we have

$$\begin{array}{ccc} \rho_{fol}\left(a,c\right) & = & \psi_{q(a)}\left(a\right)-\psi_{q(c)}\left(c\right)+\sum_{i=1}^{n+1}x_{i}^{\prime }\left(c\right)\left(x^{i}\left(a\right)-x^{i}\left(c\right)\right)\\ & = & \psi_{q(b)}\left(b\right)-\psi_{q(c)}\left(c\right)+\sum_{i=1}^{n+1}\left\{x_{i}^{\prime }\left(c\right)\left(x^{i}\left(a\right)-x^{i}\left(b\right)\right)+x_{i}^{\prime }\left(c\right)\left(x^{i}\left(b\right)-x^{i}\left(c\right)\right)\right\}\\ & = & +\mu \sum_{i=1}^{n+1}x_{i}^{\prime }\left(b\right)\left(x^{i}\left(a\right)-x^{i}\left(b\right)\right)\\ & & +\{\psi_{q(b)}\left(b\right)-\psi_{q(c)}\left(c\right)+\sum_{i=1}^{n+1}x_{i}^{\prime }\left(c\right)\left(x^{i}\left(b\right)-x^{i}\left(c\right)\right)\}\\ & = & \mu \;\rho_{fol}\left(a,b\right)+\rho_{fol}\left(b,c\right). \end{array}$$

□

**Figure 1:**
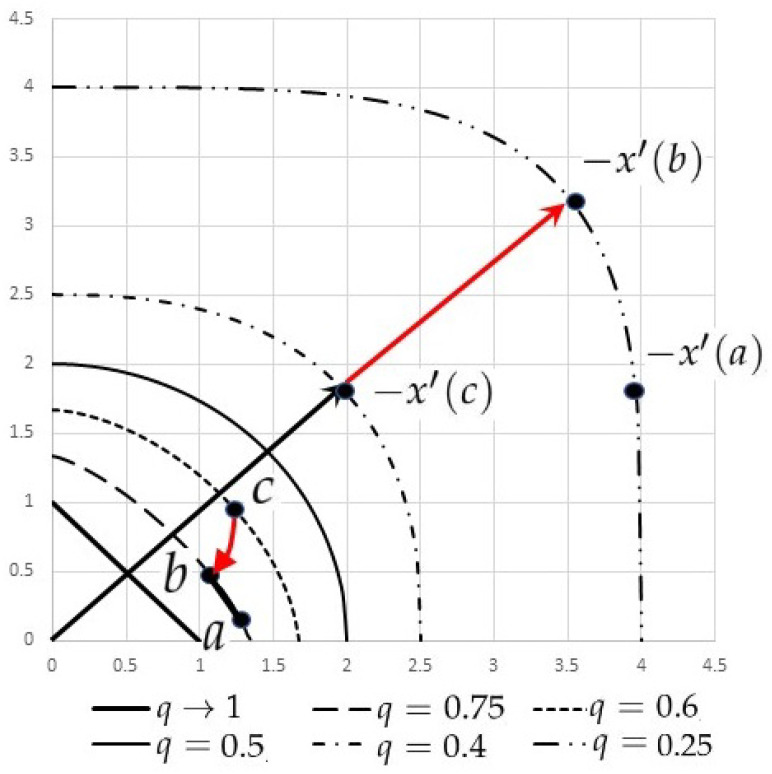
A decomposition of extended divergence $\rho_{fol}\left(a,c\right)$, graphs of the standard simplex (q→1), and deformed simplexes as q= 0.75, 0.6, 0.5, 0.4, 0.25 in $A_{+}^{2}$. For primal coordinates $a,b\in f_{0.75}\left(S^{1}\right)$, and $c\in f_{0.6}\left(S^{1}\right)$, dual coordinates satisfy $-x^{\prime }\left(a\right),-x^{\prime }\left(b\right)\in f_{0.25}\left(S^{1}\right)$, and $-x^{\prime }\left(c\right)\in f_{0.4}\left(S^{1}\right)$. The primal geodesic between *a* and *b* is orthogonal to the dual one between *b* and *c* with respect to the metric $g_{0.75}$.

See Figure 1 for a decomposition of extended divergence and graphs of deformed simplexes $f_{q}\left(S^{n}\right)$.

A decomposition similar to Equation (39) is also available on a foliation by Hessian level surfaces of one Hessian manifold [20]. Theorem 3 generalizes the previous decomposition.

Finally, we describe the gradient flow on a leaf $f_{q}\left(S^{n}\right)$ using the extended divergence.

**Theorem** **4.** *For a submanifold $\left(f_{q}\left(S^{n}\right),D,g_{q}\right)$ of $S_{fol}$, we denote by $\left\{x^{1},\cdots ,x^{n}\right\}$ an affine coordinate system on $f_{q}\left(S^{n}\right)$ such that $Ddx^{i}=0$, i=1,⋯,n, and set $g_{q\;ij}=g_{q}\left(\partial /\partial x^{i},\partial /\partial x^{j}\right)$, $\left(g_{q}^{ij}\right)={\left(g_{q\;ij}\right)}^{-1}$. For a fixed point $c\in L^{⊥}$, the gradient flow on $f_{q}\left(S^{n}\right)$ defined by*

(40) $$\frac{dx^{i}}{dt}=-\sum_{j=1}^{n}g_{q}^{ij}\frac{\partial }{\partial x^{j}}\rho_{fol}\left(a_{x},c\right),\;\;\;\;i=1,\cdots ,n,\;\;\;\;a_{x}\in f_{q}\left(S^{n}\right)$$

*converges to the unique point $b\in L^{⊥}\cap f_{q}\left(S^{n}\right)$, where $a_{x}$ is a variable point parametrized as $\left\{x^{1}\left(t\right),\cdots ,x^{n}\left(t\right)\right\}$.*

**Proof.**  By Theorem 3, for any $a_{x}\in f_{q}\left(S^{n}\right)$, there exists μ>0 such that

$$\rho_{fol}\left(a_{x},c\right)=\mu \;\rho_{fol}\left(a_{x},b\right)+\rho_{fol}\left(b,c\right),\;\;\;\;x^{\prime }\left(c\right)=\mu \;x^{\prime }\left(b\right).$$

Equation (40) is described by the dual coordinate system $\left\{x_{1}^{\prime },\cdots ,x_{n}^{\prime }\right\}$ on $f_{q}\left(S^{n}\right)$ as follows;

(41) $$\frac{dx_{i}^{\prime }}{dt}=-\mu \frac{\partial }{\partial x^{j}}\rho_{fol}\left(a_{x},b\right),\;\;\;\;i=1,\cdots ,n.$$

On $f_{q}\left(S^{n}\right)$, from Prop. 1.(i), $\rho_{fol}$ coincides with the geometric divergence $\rho_{q}$, generated by the affine immersion $\left(f_{q},E_{q}\right)$. The geometric divergence generates the dual coordinate $x_{i}^{\prime }$ such that $D^{*}dx_{i}^{\prime }=0$, i=1,⋯,n, to be derived by $x^{i}$ [19]. Then, it holds that

(42) $$\frac{dx_{i}^{\prime }}{dt}=-\mu \left(x_{i}^{\prime }\left(a_{x}\right)-x_{i}^{\prime }\left(b\right)\right),\;\;\;\;i=1,\cdots ,n,$$

and that

(43) $$x_{i}^{\prime }=x_{i}^{\prime }\left(b\right)+\left(x_{i}^{\prime }\left(a{|}_{t=0}\right)-x_{i}^{\prime }\left(b\right)\right)e^{-\mu t},\;\;\;\;i=1,\cdots ,n,$$

where ${a|}_{t=0}$ is an initial point of Equation (40). Then, the gradient flow of Equation (40) converges to $b\in L^{⊥}\cap f_{q}\left(S^{n}\right)$ following a geodesic for the dual coordinate system. □

The gradient flow similar to Equation (40) has been provided on a flat statistical submanifold [25]. The similar one on a Hessian level surface, i.e., a 1-conformally statistical submanifold, has been given in [20]. Theorem 4 generalizes the previous theorems on gradient flows.

## 7. Conclusions

This study considers a foliation of deformed probability simplexes corresponding to sets of escort distributions with *q*-parameters, for the continuous transition of α-parameters on information geometry. Since these are typical *q*-exponential families, we still need to provide details on the extended divergence and natural definition of the foliation of *q*-exponential families.

The extended divergence guides the proximity of *q*-exponential distributions with different *q*-parameters. Therefore, our theory guarantees the mathematical basis for generalizing methods of machine learning and statistical mechanics to the case of the q-distribution families when different q-parameters are mixed. The decomposition theorem is applied to the problem of the optimal choice of *q*-parameter. The application methods are open to consideration. It also remains to investigate the relationship with a new λ-duality on nonextensive statistical mechanics with mixed parameters [26,27]. 

## Data Availability

Not applicable.
